# Further result on Dirichlet-Sch type inequality and its application

**DOI:** 10.1186/s13660-017-1381-4

**Published:** 2017-05-08

**Authors:** Liquan Wan

**Affiliations:** School of Accounting, Henan University of Economics and Law, Zhengzhou, 450046 China

**Keywords:** Dirichlet-Sch type inequality, harmonic function, Schrödinger PWB solution

## Abstract

In this paper we deal with a theoretical question raised in connection with the application of Dirichlet-Sch type inequality, obtained by Huang (Int. Math. J. 27(02):1650009, 2016), which has been already applied to obtain multiplicity results for boundary value problems in several recent papers. We also discuss a particular case of it in more detail. As an application, we deduce the least harmonic majorant and log-concavity of extended subharmonic functions.

## Introduction

Let Γ be the subset of the upper half unit sphere. The set ${\mathbf {R}}_{+}\times\Gamma$ in ${\mathbf {R}}^{n}$ is called a cone. We denote it by $\mathfrak{C}_{n}(\Gamma)$, where $\Gamma\subset{\mathbf {S}}_{1}$. The sets $I\times\Gamma$ and $I\times \partial{\Gamma}$ with an interval on **R** are denoted by $\mathfrak{C}_{n}(\Gamma;I)$ and $\mathfrak{S}_{n}(\Gamma;I)$, respectively. We denote $\mathfrak {C}_{n}(\Gamma)\cap S_{R}$ and $\mathfrak{S}_{n}(\Gamma; (0,+\infty))$ by $\mathfrak {S}_{n}(\Gamma; R)$ and $\mathfrak{S}_{n}(\Gamma)$, respectively.

Furthermore, we denote by *dσ* (resp. $dS_{R}$) the $(n-1)$-dimensional volume elements induced by the Euclidean metric on $\partial{\mathfrak{C}_{n}(\Gamma)}$ (resp. $S_{R}$) and by *dw* the elements of the Euclidean volume in ${\mathbf {R}}^{n}$.

It is well known (see, *e.g.*, [2], p.41) that

1.1 $$\begin{aligned} &{\Delta^{*}\varphi(\Theta)+\lambda\varphi(\Theta)=0\quad \textrm{in } \Gamma,} \\ \\ &{\varphi(\Theta)=0\quad\textrm{on } \partial{\Gamma},} \end{aligned}$$

where $\Delta^{*}$ is the Laplace-Beltrami operator. We denote the least positive eigenvalue of this boundary value problem (1.1) by *λ* and the normalized positive eigenfunction corresponding to *λ* by $\varphi(\Theta)$, $\int_{\Gamma}\varphi^{2}(\Theta)\,dS_{1}=1$.

We remark that the function $r^{\aleph^{\pm}}\varphi(\Theta)$ is harmonic in $\mathfrak{C}_{n}(\Gamma)$, belongs to the class $C^{2}(\mathfrak{C}_{n}(\Gamma )\backslash\{O\})$ and vanishes on $\mathfrak{S}_{n}(\Gamma)$, where

$$2\aleph^{\pm}=-n+2\pm\sqrt{(n-2)^{2}+4\lambda}. $$

For simplicity we shall write *χ* instead of $\aleph^{+}-\aleph^{-}$.

For simplicity we shall assume that the boundary of the domain Γ is twice continuously differentiable, $\varphi\in C^{2}(\overline{\Gamma})$ and $\frac{\partial\varphi}{\partial n}>0$ on *∂*Γ. Then (see [3], p.7-8)

1.2 $$ \operatorname{dist}(\Theta,\partial{\Gamma})\approx\varphi(\Theta), $$

where $\Theta\in\Gamma$.

Let $\delta(P)=\operatorname{dist}(P,\partial{\mathfrak{C}_{n}(\Gamma)})$, we have

1.3 $$ \varphi(\Theta)\approx\delta(P), $$

for any $P=(1,\Theta)\in\Gamma$ (see [4]).

Let $u(r,\Theta)$ be a function on $\mathfrak{C}_{n}(\Gamma)$. For any given $r\in{\mathbf {R}}_{+}$, the integral

$$\int_{\Gamma}u(r,\Theta)\varphi(\Theta)\,d S_{1}, $$

is denoted by $\mathcal{N}_{u}(r)$, when it exists. The finite or infinite limits

$$\lim_{r\rightarrow\infty}r^{-\aleph^{+}}\mathcal{N}_{u}(r)\quad\textrm{and}\quad \lim_{r\rightarrow0}r^{-\aleph^{-}}\mathcal{N}_{u}(r) $$

are denoted by $\mathscr{U}_{u}$ and $\mathscr{V}_{u}$, respectively, when they exist.

### Remark 1

A function $g(t)$ on $(0,\infty)$ is $\mathbb{A}_{d_{1},d_{2}}$-convex if and only if $g(t)t^{d_{2}}$ is a convex function of $t^{d}$ $(d=d_{1}+d_{2})$ on $(0,\infty)$, or, equivalently, if and only if $g(t)t^{-d_{1}}$ is a convex function of $t^{-d}$ on $(0,\infty)$.

### Remark 2

$\mathcal{N}_{u}(r)$ is a $\mathbb{A}_{\aleph^{+},\gamma-1}$-convex on $(0,\infty)$, where *u* is a subharmonic function on $\mathfrak{C}_{n}(\Gamma)$ such that

1.4 $$ \limsup_{P\in\mathfrak{C}_{n}(\Gamma),P\rightarrow Q \in \partial{\mathfrak{C}_{n}(\Gamma)}}u(P)\leq c, $$

where *c* is a nonnegative number (see [5]).

The function

$$\mathbb{P}_{\mathfrak{C}_{n}(\Gamma)}(P,Q)=\frac{\partial\mathbb {G}_{\mathfrak{C}_{n}(\Gamma)}(P,Q)}{\partial n_{Q}} $$

is called the ordinary Poisson kernel, where $\mathbb{G}_{\mathfrak{C}_{n}(\Gamma)}$ is the Green function.

The Poisson integral of *g* relative to $\mathfrak{C}_{n}(\Gamma)$ is defined by

$$\mathbb{PI}_{\mathfrak{C}_{n}(\Gamma)} [g](P)=\frac{1}{c_{n}} \int_{\mathfrak{S}_{n}(\Gamma)}\mathbb {P}_{\mathfrak{C}_{n}(\Gamma)}(P,Q)g(Q)\,d\sigma, $$

where *g* is a continuous function on $\partial{\mathfrak{C}_{n}(\Gamma)}$ and $\frac{\partial}{\partial n_{Q}}$ denotes the differentiation at *Q* along the inward normal into $\mathfrak{C}_{n}(\Gamma)$.

We set functions *f* satisfying

1.5 $$ \int_{\mathfrak{S}_{n}(\Gamma)}\frac{ \vert f(t,\Phi) \vert ^{p}}{1+t^{\gamma }}\,d\sigma< \infty, $$

where $-1< p<+\infty$ and

$$\frac{-\aleph^{+}-n+2}{p}< \gamma< \frac{-\aleph^{+}-n+2}{p}+n-1. $$

Let $-1< p<+\infty$. we denote $\mathcal{A}_{\Gamma}$ the class of all measurable functions $g(t,\Phi)$ $(Q=(t,\Phi)=(Y, y_{n})\in \mathfrak{C}_{n}(\Gamma))$ satisfying the following inequality:

$$ \int_{\mathfrak{C}_{n}(\Gamma)}\frac{ \vert g(t,\Phi) \vert ^{p-1}\varphi }{1+t^{\gamma-3}}\,dw< \infty $$

and the class $\mathcal{B}_{\Gamma}$, consists of all measurable functions $h(t,\Phi)$ $((t,\Phi)=(Y, y_{n})\in\mathfrak{S}_{n}(\Gamma))$ satisfying

$$ \int_{\mathfrak{S}_{n}(\Gamma)}\frac{ \vert h(t,\Phi) \vert ^{q}}{1+t^{\gamma}}\frac{\partial\varphi}{\partial n}\,d\sigma < \infty, $$

where $q>0$.

We will also consider the class of all continuous functions $u(t,\Phi)$ $((t,\Phi)\in\overline{\mathfrak{C}_{n}(\Gamma)})$ harmonic in $\mathfrak{C}_{n}(\Gamma)$ with $u^{+}(t,\Phi)\in \mathcal{A}_{\Gamma}$ $((t,\Phi)\in\mathfrak{C}_{n}(\Gamma))$ and $u^{+}(t,\Phi)\in\mathcal{B}_{\Gamma}$ $((t,\Phi)\in\mathfrak {S}_{n}(\Gamma))$ is denoted by $\mathcal{C}_{\Gamma}$ (see [6]).

In 2015, Jiang, Hou and Peixoto-de-Büyükkurt (see [7]) obtained the following result.

### Theorem A

*Let* *g* *be a measurable function on* $\partial{T_{n}}$ *such that*

$$\int_{\partial{T_{n}}}\bigl(1+ \vert Q \vert \bigr)^{2-n} \bigl\vert g(Q) \bigr\vert \,dQ< \infty. $$

*Then the harmonic function* $\mathbb{PI}_{T_{n}}[g]$ *satisfies* $\mathbb{PI}_{T_{n}}[g](P)=o(r^{2}\sec^{n-3}\theta_{1})$ *as* $r\rightarrow\infty$ *in* $T_{n}$.

*Recently*, *Wang*, *Huang and N*. *Yamini* (*see* [8]) *generalized Theorem* A *to the conical case*.

### Theorem B

*Let* *g* *be a continuous function on* $\partial {\mathfrak{C}_{n}(\Gamma)}$ *satisfying* (1.5) *with* $p=q=1$ *and* $\gamma=\aleph^{+}+1-\aleph^{-}$. *Then*

$$\mathscr{U}_{\mathbb{PI}_{\mathfrak{C}_{n}(\Gamma)}[g]}=\mathscr {U}_{\mathbb{PI}_{\mathfrak{C}_{n}(\Gamma)}[ \vert g \vert ]}=0. $$

*The remainder of the paper is organized as follows*: *in Section* 2, *we shall give our main theorem*; *in Section* 3, *some necessary lemmas are given*; *in Section* 4, *we shall prove the main result*.

## Main result

In this section, we give the main result of this paper.

Our main aim is to give a least harmonic majorant of a nonnegative subharmonic function on $\mathfrak{C}_{n}(\Gamma)$.

### Theorem 1

*Let* *u* *be a function subharmonic in* $\mathfrak {C}_{n}(\Gamma)$ *and* $u'$ *be the restriction of* *u* *to* $\partial{\mathfrak{C}_{n}(\Gamma)}$. *If* $u'$ *satisfy* (1.5) *and* $-1\leq\mathscr{U}_{u}\leq1$ *then*

2.1 $$ u(P)\leq h_{u}(P) $$

*for any* $P=(r,\Theta)\in\mathfrak{C}_{n}(\Gamma)$, *where* $h_{u}(P)$ *is the least harmonic majorant of* *u* *on* $\mathfrak{C}_{n}(\Gamma)$ *and has the following expression*:

$$h_{u}(P)=\mathbb{PI}_{\mathfrak{C}_{n}(\Gamma)}\bigl[u'\bigr](P)+ \mathscr {V}_{u}r^{\aleph^{-}}\varphi(\Theta)+\mathscr{U}_{u}r^{\aleph ^{+}} \varphi(\Theta). $$

### Remark 3

Theorem 1 solves a theoretical question raised in connection with the application of Dirichlet-Sch type inequality, obtained by Huang (see [1]), which has been already applied to obtain multiplicity results for boundary value problems in several recent papers.

## Main lemmas

In order to prove our main result, we need the following lemmas.

### Lemma 1

see [1]

*Let* *u* *be a function subharmonic on* $\mathfrak{C}_{n}(\Gamma)$ *satisfying* (1.4). *Then the limit* $\mathscr{U}_{u}$ $(-1<\mathscr{U}_{u}\leq1)$ *exists*.

### Lemma 2

*Let* *u* *be a function subharmonic on* $\mathfrak{C}_{n}(\Gamma)$ *satisfying* (1.4) *and*

3.1 $$ \mathscr{U}_{u^{+}}\leq1\quad\textrm{and}\quad \mathscr {U}_{u^{+}}< +\infty. $$

*Then*

3.2 $$ u(r,\Theta)\leq\mathscr{V}_{u^{+}}r^{\aleph^{-}}\varphi( \Theta)+ \mathscr{U}_{u^{+}}r^{\aleph^{+}}\varphi(\Theta). $$

### Proof

Take any $(r,\Theta)\in\mathfrak{C}_{n}(\Gamma)$ and any pair of numbers $\tau_{1}$, $\tau_{2}$ $(0<\tau_{1}<r<\tau_{2}<+\infty)$. We define a boundary function on $\partial{\mathfrak{C}_{n}(\Gamma;(\tau_{1},\tau_{2}))}$ by

$$\nu(r,\Theta)= \textstyle\begin{cases} u(\tau_{i},\Theta) & \mbox{on } \{\tau_{i}\}\times\Gamma\ (i=1,2),\\ 0 & \mbox{on } [\tau_{1},\tau_{2}]\times\partial{\Gamma}. \end{cases} $$

If we denote Schrödinger PWB solution of the Dirichlet-Sch problem on $\mathfrak{C}_{n}(\Gamma;(\tau_{1},\tau_{2}))$ with *ν* by $H_{\nu}((r,\Theta);\mathfrak{C}_{n}(\Gamma;(\tau_{1},\tau_{2})))$, then we have

$$\begin{aligned} u(r,\Theta) \leq& H_{\nu}\bigl((r,\Theta); \mathfrak{C}_{n}\bigl(\Gamma;(\tau_{1},\tau_{2}) \bigr)\bigr) \\ \leq& \int_{\Gamma}u^{+}(\tau_{1},\Theta) \frac{\partial \mathbb{G}_{\mathfrak{C}_{n}(\Gamma;(\tau_{1},\tau_{2}))}((\tau_{1},\Phi ),(r,\Theta))}{\partial R}\tau_{1}^{n-1}\,dS_{1} \\ &{} - \int_{\Gamma}u^{+}(\tau_{2},\Theta) \frac{\partial \mathbb{G}_{\mathfrak{C}_{n}(\Gamma;(\tau_{1},\tau_{2}))}((\tau_{2},\Phi ),(r,\Theta))}{\partial R}\tau_{2}^{n-1}\,dS_{1}, \end{aligned}$$

which shows that (3.2) holds from (3.1). □

### Lemma 3

*Let* *g* *be a locally integrable function on* $\partial{\mathfrak{C}_{n}(\Gamma)}$ *satisfying* (1.5) *and u be a subharmonic function on* $\mathfrak{C}_{n}(\Gamma)$ *satisfying*

3.3 $$ -1\leq\liminf_{P\in\mathfrak {C}_{n}(\Gamma), P\rightarrow Q\in\partial{\mathfrak{C}_{n}(\Gamma)}}\bigl\{ u(P)- \mathbb{PI}_{\mathfrak {C}_{n}(\Gamma)}[g](P)\bigr\} \leq1 $$

*and*

3.4 $$ \liminf_{P\in\mathfrak{C}_{n}(\Gamma), P\rightarrow Q\in\partial{\mathfrak{C}_{n}(\Gamma)}}\bigl\{ u^{+}(P)-\mathbb {PI}_{\mathfrak{C}_{n}(\Gamma)}\bigl[ \vert g \vert \bigr](P)\bigr\} \leq0. $$

*Then the limits* $\mathscr{U}_{u}$ *and* $\mathscr{V}_{u^{+}}$ ($-\infty<\mathscr{U}_{u}\leq1$, $0\leq\mathscr{U}_{u^{+}}\leq+\infty$) *exist*, *and if* (3.1) *is satisfied*, *then*

3.5 $$ u(P)\leq \mathbb{PI}_{\mathfrak{C}_{n}(\Gamma)}[g](P)+\mathscr {V}_{u^{+}}r^{\aleph^{-}}\varphi(\Theta)+\mathscr {U}_{u^{+}}r^{\aleph^{+}} \varphi(\Theta) $$

*for any* $P=(r,\Theta)\in\mathfrak{C}_{n}(\Gamma)$.

### Proof

Put

$$U(P)=u(P)-\mathbb{PI}_{\mathfrak{C}_{n}(\Gamma)}[g](P)\quad\textrm {and}\quad U'(P)=u^{+}(P)- \mathbb{PI}_{\mathfrak{C}_{n}(\Gamma)}\bigl[ \vert g \vert \bigr](P) $$

on $\mathfrak{C}_{n}(\Gamma)$. From (3.3) and (3.4) we have

$$\liminf_{P\in\mathfrak{C}_{n}(\Gamma), P\rightarrow Q}U(P)\leq-1\quad \textrm{and}\quad \liminf _{P\in\mathfrak {C}_{n}(\Gamma), P\rightarrow Q}U'(P)\leq-1. $$

Hence it follows from Lemma 1 that the limits $\mathscr{U}_{U}$ and $\mathscr{V}_{U'}$ ($-1<\mathscr{U}_{U}\leq1$, $0\leq\mathscr{V}_{U'}\leq1$) exist. So Theorem B gives the existence of the limits $\mathscr{U}_{u}$, $\mathscr{V}_{u^{+}}$,

3.6 $$ \mathscr{U}_{U}=\mathscr{V}_{u}\quad\textrm{and}\quad \mathscr {U}_{U'}=\mathscr{V}_{u^{+}}. $$

Since $0\leq U^{+}(P)\leq u^{+}(P)+(\mathbb{PI}_{\mathfrak {C}_{n}(\Gamma)}[g])^{-}(P)$ on $\mathfrak{C}_{n}(\Gamma)$, it also follows from Theorem B and (3.1) that

$$\mathscr{V}_{U^{+}}\leq\mathscr{V}_{u^{+}}< \infty, $$

which together with Lemma 2 gives the conclusion. □

### Lemma 4

*Let* *g* *be a lower semi*-*continuous function on* $\partial{\mathfrak{C}_{n}(\Gamma)}$ *satisfying* (1.5) *and u be a superharmonic function on* $\mathfrak{C}_{n}(\Gamma)$ *such that*

3.7 $$ \liminf_{P\in\mathfrak{C}_{n}(\Gamma), P\rightarrow Q}u(P)\leq g(Q)+c $$

*for any* $Q\in\partial{\mathfrak{C}_{n}(\Gamma)}$ *and* *c* *is a positive number*. *Then the limit* $\mathscr{U}_{u}$ ($-1\leq\mathscr{U}_{u}\leq+1$) *exists*, *and if* $\mathscr{U}_{u}<+\infty$, *then*

$$u(P)\leq \mathbb{PI}_{\mathfrak{C}_{n}(\Gamma)}[g](P)+\mathscr{V}_{u}r^{\aleph ^{-}} \varphi(\Theta)+\mathscr{U}_{u}r^{\aleph^{+}}\varphi(\Theta) $$

*for any* $P=(r,\Theta)\in\mathfrak{C}_{n}(\Gamma)$.

### Proof

Since −*g* is upper semi-continuous function in $\partial{\mathfrak{C}_{n}(\Gamma)}$, it follows from [8], p.3, that

3.8 $$ \liminf_{P\in\mathfrak{C}_{n}(\Gamma), P\rightarrow Q}\mathbb{PI}_{\mathfrak{C}_{n}(\Gamma)}[g](P) \geq g(Q)-c. $$

We see from (3.7) and (3.8) that

$$-1\leq\limsup_{P\in\mathfrak{C}_{n}(\Gamma), P\rightarrow Q}\bigl\{ u(P)-\mathbb{PI}_{\mathfrak{C}_{n}(\Gamma)}[g](P) \bigr\} \leq1, $$

which gives (3.3). Since *g* and *u* are positive, (3.4) also holds. Lemma 4 is proved. □

### Lemma 5

*Let* *u* *be a subharmonic function in* $\overline {\mathfrak{C}_{n}(\Gamma)}$ *such that* $u'=u|\partial{\mathfrak{C}_{n}(\Gamma)}$ *satisfies* (1.5). *Then* $\mathbb{PI}_{\mathfrak{C}_{n}(\Gamma)}[u'](P)\leq h(P)$ *on* $\mathfrak{C}_{n}(\Gamma)$, *where* $h(P)$ *is the any harmonic majorant of* *u* *on* $\mathfrak{C}_{n}(\Gamma)$.

### Proof

Take any $P'=(r',\Theta')\in \mathfrak{C}_{n}(\Gamma)$. Let *ϵ* be any positive number. In the same way as in the proof of Lemma 2, we can choose *R* such that

3.9 $$ \int_{\mathfrak{S}_{n}(\Gamma;(R,\infty))}\mathbb{P}_{\mathfrak {C}_{n}(\Gamma)}\bigl(P',Q \bigr)u'(Q)\,d\sigma< \frac{\epsilon}{2}. $$

Further, take an integer *j* $(j>R)$ such that (see [7])

3.10 $$ \int_{\mathfrak{S}_{n}(\Gamma;(0,R))}\frac{\partial\Gamma_{j}(P',Q) }{\partial n_{Q}}u'(Q)\,d\sigma< \frac{\epsilon}{2}. $$

Since

$$\int_{\mathfrak{S}_{n}(\Gamma;(0,R))}\frac{\partial \mathbb{G}_{\mathfrak{C}_{n}(\Gamma;(0,j))}(P,Q) }{\partial n_{Q}}u'(Q)\,d\sigma\leq H_{u}\bigl(P;\mathfrak{C}_{n}\bigl(\Gamma;(0,j)\bigr)\bigr) $$

for any $P\in\mathfrak{C}_{n}(\Gamma;(0,j))$, we have from (3.9) and (3.10)

3.11 $$\begin{aligned} &{\mathbb{PI}_{\mathfrak{C}_{n}(\Gamma)}\bigl[u'\bigr] \bigl(P'\bigr)-H_{u}\bigl(P';\mathfrak {C}_{n}\bigl(\Gamma;(0,j)\bigr)\bigr)} \\ &{\quad\leq \int_{\mathfrak{S}_{n}(\Gamma;(0,R))}\frac{\partial \Gamma_{j}(P',Q) }{\partial n_{Q}}u'(Q)\,d\sigma} \\ &{\qquad {}+\frac{1}{c_{n}} \int_{\mathfrak{S}_{n}(\Gamma;(R,\infty))}\mathbb {P}_{\mathfrak{C}_{n}(\Gamma)}\bigl(P',Q \bigr)u'(Q)\,d\sigma} \\ &{\quad < \epsilon.} \end{aligned}$$

Here note that $H_{u}(P;\mathfrak{C}_{n}(\Gamma;(0,j)))$ is the least harmonic majorant of *u* on $\mathfrak{C}_{n}(\Gamma;(0,j))$ (see [9], Theorem 3.15). If *h* is a harmonic majorant of *u* on $\mathfrak{C}_{n}(\Gamma)$, then

$$H_{u}\bigl(P';\mathfrak{C}_{n}\bigl( \Gamma;(0,j)\bigr)\bigr)\leq h\bigl(P'\bigr). $$

Thus we obtain from (3.11)

$$\mathbb{PI}_{\mathfrak{C}_{n}(\Gamma)}\bigl[u'\bigr]\bigl(P' \bigr)< h\bigl(P'\bigr)+\epsilon, $$

which gives the conclusion of Lemma 5. □

## Proof of Theorem 1

Let *P* be any point of $\mathfrak{C}_{n}(\Gamma)$ and *ϵ* be any positive number. By the Vitali-Carathéodory theorem with respect to the Schrödinger operator (see [10], p.56), there exists a lower semi-continuous function $g'(Q)$ on $\partial{\mathfrak {C}_{n}(\Gamma)}$ such that

4.1 $$ u'(Q)\leq g'(Q) $$

and

4.2 $$ \mathbb{PI}_{\mathfrak{C}_{n}(\Gamma)}\bigl[g'\bigr](P)< \mathbb{PI}_{\mathfrak {C}_{n}(\Gamma)}\bigl[u'\bigr](P)+\epsilon. $$

Since

$$\lim_{P\in\mathfrak{C}_{n}(\Gamma), P\rightarrow Q}u(P)\leq u'(Q)\leq g'(Q) $$

for any $Q\in\partial{\mathfrak{C}_{n}(\Gamma)}$ from (4.1), it follows from [1], Lemma 2.1, that the limits $\mathscr {U}_{u}$ and $\mathscr{U}_{u}$ exist, and if $-1\leq\mathscr{U}_{u}<1$ and $-1\leq\mathscr{V}_{u}<1$, then

4.3 $$ u(P)\leq\mathbb{PI}_{\mathfrak{C}_{n}(\Gamma)}\bigl[g'\bigr](P)+ \mathscr {V}_{u}r^{\aleph^{-}}\varphi(\Theta)+\mathscr{U}_{u}r^{\aleph ^{+}} \varphi(\Theta). $$

Hence we see from (4.2) and (4.3) that (2.1) holds.

Next we call the least harmonic majorant of *u* on $\mathfrak{C}_{n}(\Gamma)$: $h_{u}(P)$. Set $h''(P)$ is a Schrödinger harmonic function in $\mathfrak{C}_{n}(\Gamma)$ such that (see [7])

4.4 $$ u(P)\leq h''(P)+\epsilon. $$

Put

$$h^{\ast}(P)=h_{u}(P)-h''(P)\quad\mbox{on }\mathfrak{C}_{n}(\Gamma). $$

It is easy to see that

$$h^{\ast}(P)\leq h_{u}(P). $$

It follows from Theorem B that $\mathscr{V}_{{h^{\ast}}^{+}}<+\infty$. Further, from Lemma 5 we see that

$$\limsup_{P\in\mathfrak{C}_{n}(\Gamma), P\rightarrow Q}h^{\ast}(P)=\liminf_{P\in\mathfrak{C}_{n}(\Gamma), P\rightarrow Q} \bigl\{ \mathbb{PI}_{\mathfrak{C}_{n}(\Gamma)}\bigl[u'\bigr](P)-h''(P) \bigr\} \leq-1. $$

From Theorem B and (4.4) we know

$$\mathscr{V}_{h^{\ast}}=\mathscr{V}_{h_{u}}-\mathscr{V}_{h''}= \mathscr {V}_{u}-\mathscr{U}_{h''}\leq\mathscr{U}_{u}- \mathscr{U}_{u}=0. $$

We see from Lemma 2 that $-1\leq h^{\ast}(P)\leq\epsilon$ on $\mathfrak{C}_{n}(\Gamma)$, which shows that $h_{u}(P)$ is the least harmonic majorant in $\mathfrak{C}_{n}(\Gamma)$. Theorem 1 is proved.

## Conclusion

In this article, we dealt with a theoretical question raised in connection with the application of Dirichlet-Sch type inequality. Additionally, we discussed a particular case of it in more detail. As applications, we deduced the least harmonic majorant and log-concavity of extended subharmonic functions.
